# Adolescents’ Digital Technology Use, Emotional Dysregulation, and Self-Esteem: No Evidence of Same-Day Linkages

**DOI:** 10.1007/s42761-024-00282-w

**Published:** 2024-11-27

**Authors:** Madison E. Taylor, Stephen M. Schueller, Michael A. Russell, Rick H. Hoyle, Candice L. Odgers

**Affiliations:** 1 Department of Psychological Science, University of California, Irvine, Irvine, CA USA; 2 Department of Biobehavioral Health, The Pennsylvania State University, University Park, PA USA; 3 Department of Psychology and Neuroscience, Duke University, Durham, NC USA; 4 Social Science Research Institute, Duke University, Durham, NC USA

**Keywords:** Technology use, Emotional dysregulation, Self-esteem, Adolescents, Ecological momentary assessment, Gender differences

## Abstract

Concerns regarding the potential negative impacts of digital technology use on youth mental health and well-being are high. However, most studies have several methodological limitations: relying on cross-sectional designs and retrospective reports, assessing technology use as an omnibus construct, and focusing on between- instead of within-person comparisons. This study addresses these limitations by prospectively following young adolescents (*n* = 388) over a 14-day ecological momentary assessment study to test whether adolescents’ digital technology use is linked with self-reported emotional dysregulation and self-esteem and whether these relationships are stronger for adolescent girls than boys. We found no evidence that adolescents experienced higher emotional dysregulation (*b* = − .02; *p* = .07) and lower self-esteem (*b* = .004; *p* = .32) than they normally do on days where they use more technology than they normally do (within-person). Adolescents with higher average daily technology use over the study period did not experience lower levels of self-esteem (between-person, *b* = − .02; *p* = .13). Adolescents with higher average daily technology use across the two-week period did report higher levels of emotional dysregulation (*p* = .01), albeit the between-person relation was small (*b* = .08). There was no evidence that gender moderated the associations, both between and within adolescents (*b*s = − .02–.13, *p* = .06 − .55). Our findings contribute to the growing counter-narrative that technology use does not have as large of an impact on adolescents’ mental health and well-being as the public is concerned about.

**Supplementary Information:**

The online version contains supplementary material available at 10.1007/s42761-024-00282-w.

Scholars, parents, and policymakers have expressed concerns regarding the potential negative impact of digital technology use (i.e., “technology use”) on youth mental health and well-being, especially after a recent advisory from the United States Surgeon General (Office of the Surgeon General, 2023; Twenge et al., 2018). This includes beliefs that increases in daily technology use have led to increases in negative moods, depression and anxiety, and worsened self-esteem (Haidt & Allen, 2020). However, meta-analytic reviews and large longitudinal studies testing the relation between technology use and youth mental health support a different story. These studies suggest the relation between technology use and adolescent mental health (e.g., anxiety, depression) is null, or, if negative, is small (Hancock et al., 2022; Valkenburg et al., 2022). However, several methodological limitations of previous studies impede our ability to truly decipher the relation between these constructs.

One such limitation is the use of cross-sectional, retrospective self-reports from adolescents (Odgers et al., 2020). While such designs can answer questions about between-person correlations (i.e., “Do youth who use more technology on average also have higher mental health problems?”), they cannot answer questions about within-person relations (i.e., “When an adolescent uses more technology than they normally do, do they experience more mental health problems than usual?”). Longitudinal studies that examine within-person relations are rare (Valkenburg et al., 2022). Of those that exist, within-person associations between digital technology use and indicators of adolescent mental health have been null or negative, but small (Beeres et al., 2021; Boer et al., 2022; Boers et al., 2019; Coyne et al., 2020). However, these studies tend to lack design elements, such as daily repeated measures, that allow for us to examine this relation on a day-to-day basis.

The bulk of prior research on technology use and adolescent mental health has also rarely delineated between different types of technology use. Although adolescents use technology for a variety of activities, it is common for studies to only use an aggregate measure of time spent using technology or social media (Salen Tekinbaş et al., 2023). In reality, adolescents may use technology for a variety of reasons, including to complete necessary schoolwork, connect with friends, entertain themselves (e.g., watching videos, reading posts, playing games), or create online content themselves. Delineating between these types of use is crucial, as how adolescents use technology influences its impact on their mental health (Verduyn et al., 2017). For example, passive Facebook use (i.e., minimal effort engagement) has predicted increases in depressed moods for adolescent girls, while active Facebook use (e.g., higher engagement) among friends has predicted decreases in depressed moods (Frison & Eggermont, 2016).

Technology use may also not impact all youth equally. Adolescents have reported feeling better, feeling worse, or no changes to their mental health when using online platforms such as social media (Rideout & Fox, 2018). Despite this, prior research has focused on characterizing the relation between technology use and adolescent well-being using “one-size-fits-all” approaches (Odgers & Jensen, 2020). Going forward, the field may benefit from investigating heterogeneity in this relation across adolescents via assessing for moderators that distinguish which adolescents may be more vulnerable (or benefit the most) from technology use. Previous literature has suggested that adolescent girls may have stronger relations between using technology and worsened mental health (Viner et al., 2019; Kelly et al., 2018).

Additionally, most research on technology use and adolescent mental health overly focuses on symptoms of specific disorders, such as anxiety and depression, rather than other aspects of adolescent well-being, such as emotional dysregulation and self-esteem. Emotional dysregulation is a transdiagnostic factor commonly experienced by adolescents as they navigate changing environmental, social, and internal “landscapes” (De Berardis et al., 2020). While many adolescents learn to manage their emotions over time, adolescents who do not may be more vulnerable to developing psychiatric conditions (Bullis et al., 2019; De Berardis et al., 2020). This skill, deemed emotional regulation, has been linked to technology use (Gioia et al., 2021). Self-esteem increases across adolescence and into adulthood (e.g., Wagner et al., 2013), yet adolescents who struggle with low self-esteem have poorer mental health outcomes compared to their peers (Isomaa et al., 2013). Self-esteem has been linked to technology use in youth, with adolescents who demonstrate more use of certain types of technology (i.e., social media) reporting lower self-esteem (Mann et al., 2004; Valkenburg et al., 2021).

Self-esteem and emotional dysregulation might fluctuate more daily than anxiety and depression (Mann et al., 2004; Paulus et al., 2021), potentially making them more vulnerable to being influenced by daily technology use. Prior work found that emotional dysregulation may be heightened (up to 20% of a standard deviation higher in a study by McNamee et al., 2021) and self-esteem may be lower (*r* = − .17; Woods & Scott, 2016) in youth who use technology more than their peers. Research on within-person relations for these variables is limited; one study using within-person analyses found that while many adolescents showed no relations between daily technology use and self-esteem, more adolescents showed a negative person-specific relation rather than a positive one (Valkenburg et al., 2021). However, different types of technology use and potential moderators such as gender were not explored in this work.

In the present study, we addressed these gaps by testing whether adolescents’ daily technology use is associated with same-day emotional dysregulation and self-esteem. Additionally, we conducted exploratory analyses to identify if certain types of technology use were more strongly associated with these outcomes. We also investigated if adolescent girls experienced stronger between-person and within-person relations than adolescent boys, as several studies have found that adolescent girls who use more technology on average have worse mental health and well-being outcomes, but not boys (Steinsbekk et al., 2021). Our use of data from a 14-day EMA period allowed us to examine both within- and between-person effects, minimize retrospective recall for technology use, and assess *how* adolescents are using technology with a nuanced measure of types of technology use.

We hypothesized the following:

1. Adolescents with higher average daily technology use across the two-week period will have higher levels of emotional dysregulation and lower levels of self-esteem (cross-sectional associations at the between-person level).
2. On days when adolescents use more technology, they will experience more emotional dysregulation and lower self-esteem than they normally do (within-person level).
3. Girls will be more likely to experience negative associations between daily technology use and self-esteem and positive associations between technology use and emotional dysregulation than boys at both the between- and within-person levels.

## Method

### Sample and Procedure

Data came from the RAISE Study, a longitudinal study of adolescent health behaviors. The sample was selected to be demographically representative of the population of children enrolled in grades 3–6 in North Carolina Public Schools during the 2011–2012 school year based on administrative data from the North Carolina Department of Public Instruction (NCDPI). Representativeness was based on economic disadvantage, gender, and ethnicity (see Rivenbark et al. (2019) for a full sample and study description). Participants completed an initial T1 Adolescent Survey (*N* = 2104) between April and August 2015, at which time participants were enrolled in grades 5–8 and ranged in age from 9 to 15 (*M* = 12.36, *SD* = 1.12). A representative subsample of 395 early to mid-adolescents were recruited to participate in a Home Visit and a 14-day EMA between April 2016 and February 2017.

Of the 395 adolescents invited to complete the EMA period, 388 adolescents completed at least two surveys and are included in the analyses. Sixty-eight percent of the adolescents provided data on at least 80% of the 14 possible days (with an average of 3.05 missing days per adolescent). The age of adolescents in this sample ranged from 10 to 17 years (*M* = 13.4, *SD* = 1.14). The sample had an almost equal gender distribution, with 49.7% of the participants identifying as girls. A little over half of the sample identified as White non-Hispanic (60.9%), with other participants identifying as Black non-Hispanic (19.4%), Hispanic of any race (13.1%), and other races/ethnicities (i.e., Asian, American Indian, Native Hawaiian/Pacific Islander, multiracial, and those who did not report a racial/ethnic group; 6.6%). A total of 31.1% of the sample was classified as persistently economically disadvantaged based on being eligible for free or reduced lunch within the school districts from 2009 to 2016 (i.e., fell at or below 175% of the federal poverty level cutoff). Informed consent was obtained from all research participants.

Adolescents were sent three surveys a day: morning, afternoon, and evening. In all three surveys, adolescents reported on their emotional dysregulation and irritability, as well as self-esteem. Adolescents reported their daily technology use in the evening survey. All moderators were taken from the participating adolescents’ responses to a baseline survey conducted in 2015. All procedures, protocols, and measures were approved by the Duke University Institutional Review Board for the RAISE study (approval #D0396).

### Measures

#### Primary Measures

##### Emotional Dysregulation and Irritability

Adolescents reported on their emotional dysregulation and irritability in the morning, afternoon, and evening surveys using a four-item Irritability and Emotional Dysregulation Scale. The four items were adapted from the Affect Regulation Checklist (Moretti, 2003) and the Brief Self-Control Scale (Tangney et al., 2004). These items asked adolescents to rate their ability to concentrate (“I’m having a hard time concentrating or focusing”), impulsivity (“I’ve been doing or saying things without thinking first”), irritability (“Even the little things are getting on my nerves”), and inability to control emotions (“I’ve been having a hard time controlling my emotions”) on a Likert-type scale ranging from 1 (“not at all”) to 5 (“very”). Adolescents’ responses to all four items were added together to create a summary score ranging from 4 to 20. The ICC for this measure was .68.

##### Self-Esteem

Adolescents reported on their self-esteem in the morning, afternoon, and evening surveys using a single item asking “How do you feel about yourself right now?.” Response options range from 1 (“really bad”) to 5 (“really good”) on a Likert-type scale. The ICC for this measure was .66.

##### Daily Technology Use

Adolescents reported their daily technology use during the evening survey. Participants responded to four questions that began with the stem “How many hours did you spend online or on your phone…” followed by one of four activity types. These activity types included how many hours a day they spent online or on their phone doing schoolwork, talking to others or sending messages (i.e., social connection), entertainment (such as browsing social media, watching videos, or playing games), and creating content (such as posting on social media and creating videos). Participants wrote their answers in a numeric fill-in box. The responses to those four items were added together to create a score for total daily hours of technology use. The ICC for the composite daily technology use variable was .60.

#### Moderators

##### Gender

Adolescents were asked if they identify as a girl or a boy (i.e., “Are you a girl or a boy?”) in a demographic questionnaire. In the dataset, “Boy” was coded as 0, and “Girl” was coded as 1.

### Analytic Plan

All analyses were conducted in R (version 2022.07.2). The analyses for this study were pre-registered on Open Science Framework prior to analyzing the data (10.17605/OSF.IO/4J2P8). As described in the pre-registration, each adolescent’s self-esteem and composite emotional dysregulation ratings for the day were averaged together to create daily mean self-esteem and daily mean emotional dysregulation variables. The range of responses for the total daily technology use variable was inspected, revealing 120 out of 4094 days had daily technology use averages that were impossible (i.e., above 24 h a day). These responses were removed from the dataset, leaving 3974 days in the analytic sample.

To test the stated hypotheses, linear mixed-effect models were used that included both fixed and random effects in the same model. These models were generated using the *nlme* package in R (Pinheiro et al., 2023). Two separate sets of models were run where daily technology use was specified as the predictor, and self-esteem and emotional dysregulation were specified as the outcomes. We then tested for the moderation of the associations between technology use and the outcomes by gender. If the between-person or within-person effects for daily technology use or any interaction terms in the model were statistically significant, exploratory analyses were conducted to probe which types of technology use were contributing to the association(s). To do this, the predictor variable for each model was replaced with one of the four component types of technology use (i.e., technology use for schoolwork, social connection, entertainment, and content creation) and the models were re-run.

Models were fit both with and without a first-order autoregressive structure using the *nlme* package (Pinheiro et al., 2023). If the statistical significance of the within-person or between-person results differed between the models, a chi-squared test was run using the anova() command (R Core Team, 2023) to determine if there was significant autocorrelation in the residuals. If the results of the chi-squared test were statistically significant, the model with a first-order autoregressive structure was interpreted. Additionally, the residuals were assessed for non-normality, and when they were not normally distributed, models were recreated using the *lme4* package (Bates et al., 2015). They were then bootstrapped with 2000 iterations using the *boot* package in R (Canty & Ripley, 2022). The resulting 95% confidence intervals for the coefficients were interpreted.

Finally, we tested whether extreme values of daily technology use may have influenced our findings by removing all values above and below two standard deviations of the mean using the *dplyr* package (Wickham et al., 2023) and re-running the models. We also tested whether missing data patterns may have biased our results by dividing participants into quartiles based on the percentage of days where technology use was not reported and tested to see if results differed across these groups.

## Results

Descriptive statistics for the variables (means, standard deviations, skew, kurtosis, ICCs, and percentage of the sample comprising each categorical variable) are found in Table 1. Correlations between variables used in analyses are found in Table 2.

**Table 1:** Descriptive statistics for variables used in analyses

Variable name	*N*	*M*	*SD*	SK	*K*	*SE*	ICC
Total daily technology use (hours)	377	4.06	3.49	1.84	4.97	.18	-
Daily technology use for schoolwork (hours)	380	.76	1.12	5.32	49.7	.06	-
Daily technology use for social connection (hours)	378	1.34	2.37	7.44	77.6	.12	-
Daily technology use for entertainment (hours)	380	2.33	10.6	18.43	349	.12	-
Daily technology use for content creation (hours)	379	.35	.65	3.25	14.39	.03	-
Daily emotion dysregulation (4–20 Likert scale)	377	5.55	2.25	2.10	4.98	.12	.68
Daily self-esteem (1–5 Likert scale)	388	3.95	.78	− .26	− 1.04	.04	.66
Gender*	388	.50	.50	.01	− 2.01	.03	-

*0 = Boy, 1 = Girl

**Table 2:** Correlation matrix between variables used in analyses

Variable name	Total daily technology use (hours)	Daily technology use for schoolwork	Daily technology use for social connection	Daily technology use for entertainment	Daily technology use for content creation	Daily emotional dysregulation	Daily self-esteem	Gender
Total daily technology use	1.00	-	-	-	-	-	-	-
Daily technology use for schoolwork	.48**	1.00	-	-	-	-	-	-
Daily technology use for social connection	.73**	.12**	1.00	-	-	-	-	-
Daily technology use for entertainment	.73**	.05*	.07**	1.00	-	-	-	-
Daily technology use for content creation	.50**	.16**	.20**	.22**	1.00	-	-	-
Daily emotional dysregulation	.05*	.07*	.05*	.03	.08**	1.00	-	-
Daily self-esteem	− .04*	− .04*	− .02	− .01	.01	− .35**	1.00	-
Gender	.06**	.03*	.03	− .03	.01	.09**	− .16**	1.00

*p < .05, **p < .001

### Emotional Dysregulation

#### Within-Person

As shown in Table 3, we found no evidence of a relation between daily technology use and emotional dysregulation on the within-person level.

**Table 3:** Multilevel models of daily associations between technology use, emotional dysregulation, and self-esteem

Predictors	Emotional dysregulation (3,959 days, *N* = 376)				Self-esteem (3,974 days, *N* = 377)			
	Estimate	*SE*	95% CI	*p*	Estimate	*SE*	95% CI	*p*
Fixed								
Intercept	5.57	.11	(5.35, 5.80)	** < .001**	3.94	.04	(3.86, 4.02)	** < .001**
Daily tech hours within-person	− .02	.01	(− .05, .001)*	.07	.004	.004	(− .004, .01)	.32
Average tech hours (between person)	.08	.03	(.02, .15)**	**.01**	− .02	.01	(− .04, .01)	.13
Random								
Within-person residual variance (σ2)	.01				.001			
Between-person residual variance (τ00)	4.51				.56			
ICC	1.00				1.00			

No differences in statistical significance were found between models above and models with first-order autoregressive structure. Results also held across models when controlling for school attendance. The pattern of findings for the between-person emotional dysregulation analyses held when each item on the index was analyzed separately, apart from Item 1 (*p* = .11). *Boot CI, (− .05, .001); ** Boot CI, (.02, .14)

#### Between-Person

Adolescents who reported more daily technology use, on average, across the 14-day period also report higher levels of emotional dysregulation (between person: (*b* = .08, 95%CI (.02, .15), *β* = .12, *p* = .01; see Table 3). Every one-hour increase in daily technology use above the group average predicted a .08 unit increase in emotional dysregulation. Exploratory analyses were run to probe the relation between daily technology use and emotional dysregulation (see Supplemental File 1 Tables 1a-d). A statistically significant between-person association was detected in 4 of the 4 models. Adolescents reported higher levels of emotional dysregulation also reported, on average, more daily technology use for schoolwork (*b* = .38, 95% CI (.19, .58), *β* = .19, *p* < .001), social connection (*b* = .10, 95% CI (.003, .20), *β* = .10, *p* = .04), entertainment (*b* = .03, 95% CI (.01, .06), *β* = .10, *p* = .01), and content creation (*b* = .52, 95% CI (.19, .82), *β* = .15, *p* = .002).

#### Gender Moderation

Gender positively predicted emotional dysregulation (*b* = .49, 95% CI (.05, .93), *β* = .21, *p* = .03; see Table 4), with adolescent girls on average scoring .49 points higher on the emotional dysregulation scale than adolescent boys. There was no evidence that gender moderated the within-person associations between daily technology use and emotional dysregulation. After adding gender into the model, all between-person and within-person linkages between daily technology use and emotional dysregulation were no longer statistically significant. While initial models showed a significant interaction effect between daily technology use and gender for emotional dysregulation at the between-person level (*b* = .13, 95% CI (.002, .26), *β* = .19, *p* = .045), this interaction was also no longer significant after accounting for statistically significant autocorrelation in the residuals (*b* = .12, 95% CI (− .01, .25), *β* = .18, *p* = .06).

**Table 4:** Multilevel models of daily associations between technology use, emotional dysregulation, and self-esteem with gender moderation

Predictors	Emotional dysregulation × gender (3,959 days, *N* = 376)					Self-esteem × gender (3,974 days, *N* = 377)			
	Estimate	*SE*	95%CI	*p*		Estimate	*SE*	95% CI	*p*
Fixed									
Intercept	5.31	.16	(5.00, 5.62)	** < .001**		4.10	.06	(4.00, 4.21)	** < .001**
Daily tech hours within-person	− .01	.02	(− .05, .02)*	.42		− .0003	.01	(− .01, .01)	.95
Average tech hours (between person)	.01	.05	(− .07, .10)**	.74		.001	.02	(− .03, .03)	.96
Gender	.49	.22	(.05, .93)***	**.03**		− .31	.08	(− .47, − .16)	** < .001**
Tech X gender within-person	− .02	.03	(− .06, .03)****	.55		.01	.01	(− .01, .02)	.33
Tech X gender between-person	.13	.06	(.002, .26)*****	**.045**		− .03	.02	(− .08, .01)	.18
Random									
Within-person residual variance (σ2)	.01					.001			
Between-person residual variance (τ00)	4.42					.54			
ICC	1.00					1.00			

No differences in statistical significance were found between models above and models with first-order autoregressive structure for self-esteem. Differences in emotion dysregulation with first-order autoregressive structure are described in text. *Boot CI, (− .05, .02), ** (− .08, .10), *** (.05, .94) − **** (− .06, .03), ***** (.01, .27)

To provide further clarity to our interpretation of the above, we created two different versions of our dataset, one with only girl participants and one with only boy participants, and re-ran the emotional dysregulation model in each dataset with first-order autoregressive structure included. The within-person findings were statistically non-significant in both the boy and girl datasets. In the dataset with only adolescent girls, girls who used more technology on average over the 14-day study period had experienced greater emotional dysregulation (*b* = .14, 95% CI (.03, .24), *β* = .18, *p* = .01). There was no relation between daily technology use and emotional dysregulation in the dataset with only adolescent boys (*b* = .01, 95% CI (− .07, .08), *β* = .01, *p* = .85).

### Self-Esteem

#### Within-Person

We found no evidence of a relation between daily technology use and self-esteem on the within-person level.

#### Between-Person

Self-esteem was not associated with adolescents’ average technology use across the study period. In other words, adolescents who reported more daily technology use, on average, across the 14-day period did not report lower levels of self-esteem.

#### Gender Moderation

As shown in Table 4, gender negatively predicted self-esteem (*b* = − .31, 95% CI (− .47, − .16), *β* = − .41, *p* < .001). On average, adolescent girls scored .31 points lower on the self-esteem scale than adolescent boys. There was no evidence that gender moderated the within-person or between-person associations between daily technology use and self-esteem.

### Sensitivity Analyses

#### Examining the Impact of Extreme Values of Daily Technology Use

The 4 inferential and 4 exploratory models were re-run with daily technology responses greater than two standard deviations above the mean technology use (i.e., 11.8 h) removed. The results of the 8 models can be found in Supplemental File 2 Tables 2a-f. Results held across all of the models after excluding extreme values, with the exception that, after bootstrapping, daily technology use for social connection was no longer a statistically significant predictor of emotional dysregulation between-person (95% CI_boot_ (− .005, .24)).

#### Examining the Impact of Missing Data

On average, participants were missing 3 responses for total daily technology use (*M* = 3.05, *SD* = 2.95). Quartile 1 had an average of .58 missing days (*M* = .58, *SD* = .49), Quartile 2 had an average of 2 missing days (*M* = 2, *SD* = 0), Quartile 3 had an average of 3.36 missing days (*M* = 3.36, *SD* = .48), and Quartile 4 had an average of 7.52 missing days (*M* = 7.49, *SD* = 2.76).

Results of the four models with missing data as a moderator can be found in Supplemental File 3 Tables 3a-b. A statistically significant interaction effect between daily technology use and missing data was detected in only one of the four models. For adolescents in Quartile 4 (i.e., the highest missing days), the within-person effect of daily technology use on emotional dysregulation was more negative when compared to individuals in Quartile 1 (i.e., the lowest missing days) (*b* = − .08, 95% CI (− .16, − .002),* p* = .04).

## Discussion

Despite the common narrative that technology use leads to worsened mental health and well-being in adolescents, our study finds little evidence that this is the case regarding emotional dysregulation and self-esteem. At the within-person level, adolescents did not experience higher emotional dysregulation nor lower self-esteem on days where they used more technology than normal. This suggests that, after controlling for external factors in each of the adolescents’ lives, there is no relation between technology use and our outcomes. At the between-person level, adolescents with higher average daily technology use reported higher levels of emotional dysregulation, but not lower levels of self-esteem. For every additional hour above average daily technology use, adolescents scored less than a tenth of a point higher on the emotional dysregulation measure (.08 points). As such, adolescents would need to use technology for 12 additional hours to raise their scores by 1 point on a 16-point scale. Even considerably more technology use has little meaningful impact on emotional dysregulation.

Our initial hypotheses were not supported by our findings. Although some prior between-person studies have found that emotional dysregulation and self-esteem were worse in youth who used more technology than their peers (McNamee et al., 2021; Woods & Scott, 2016), our results showed all between-person relations were small at best and within-person relations were null when examining their relation to digital technology use. Instead, our results resemble another study drawing from our same dataset, which found adolescents’ depressive and anxiety symptoms were not worse on days when they reported spending more time using technology (Jensen et al., 2019). Therefore, emotional dysregulation and self-esteem did not yield different outcomes compared to symptoms of mental disorders, despite our belief that these constructs may be more susceptible to the effects of technology use because of their potential for greater daily fluctuations (Mann et al., 2004; Paulus et al., 2021).

### Types of Technology Use

A common criticism of research on technology use and youth mental health is that measures often combine different types of use (Orben, 2020). We assessed four different types of technology use: schoolwork, social connection, entertainment, and content creation. Our exploratory analyses suggested all types contributed to this relation, albeit at different levels of magnitude. Content creation and schoolwork had the largest magnitude relations. It is worth noting that these relations do not suggest causality and so increased technology use in these domains might reflect something of the adolescent. For example, the relation between technology use for schoolwork and emotional dysregulation may be driven by adolescents’ abilities to concentrate or focus. Adolescents who are struggling to concentrate or focus may take longer to complete schoolwork than their peers. Our findings for content creation are seemingly surprising, given that active social media use, which includes content creation, has been suggested to benefit adolescent well-being (Thorisdottir et al., 2019). However, adolescents who create content online may be more likely to encounter negative interactions with others, which might impact emotional dysregulation (Salen Tekinbaş et al., 2023). Given the exploratory nature of these findings, we would recommend future work to further investigate these constructs to better understand their relation with emotional dysregulation.

### Gender Moderation

Our findings for the impact of gender on the relation between technology use, emotional dysregulation, and self-esteem are more challenging to interpret. Adolescent girls did not have stronger within-person relations between daily technology use and self-esteem than adolescent boys. Furthermore, gender was not a significant moderator of the between-person relation between average daily technology use and emotional dysregulation. However, including gender in our model eliminated the main effect of daily technology use and a significant relation on the between-person level was found in the dataset with only adolescent girls (but not boys), suggesting that girls’ relation between daily technology use and emotional dysregulation seems to be driving the finding. As within-person analyses, unlike between-person analyses, allow adolescents to act as their own control, it is possible that other aspects of being an adolescent girl may result in greater technology use and greater emotional dysregulation (e.g., developmental focus on interpersonal relationships; Salen Tekinbaş et al., 2023). To draw firmer conclusions, more research on technology use and well-being variables in samples specifically selected to untangle gender differences is necessary.

### Limitations, Strengths, and Implications

This study has the following limitations. First, technology use within a given day was self-reported by adolescents retrospectively at night, presenting the possibility of bias via adolescents’ inaccurate recall (albeit likely less than in studies which use even longer reporting delays). This once-daily assessment of technology use also prevented us from investigating the directionality of the effect for the relationship between technology use and our outcomes on a given day. Similarly, we cannot establish causality for the significant relation between daily technology use and emotional dysregulation. Additionally, even though we provided examples for what the types of technology use might include (e.g., entertainment may include “browsing social media, watching videos, playing games”), adolescents’ subjective interpretations of what is included within each given type likely influenced their reporting. This style of reporting also does not account for adolescents who may be using multiple types of technology use as once (e.g., browsing social media feeds to send posts to friends). This makes it difficult to pinpoint which specific activities are contributing to our exploratory results. Future studies may benefit from assessing adolescents’ use of specific online elements (e.g., direct messages) and the content they engage with (e.g., privateness/publicness) via self-report to identify which aspects are contributing to youth mental health and well-being (Parry et al., 2022). Supplementing adolescents’ self-reports with passive data collection, such as device or platform usage logs, could provide an additional means to observe and triangulate their daily activities on technology. Collaborations with technology companies and platforms or user-centric data donation paradigms may be useful avenues to explore for collecting this information (Ohme et al., 2023). Moreover, data collection for this study took place in 2015–2016, where the technological landscape may have been less pervasive and immersive than the “metaverse-like” spaces youth encounter today (Salen Tekinbaş et al., 2023). This may limit the generalizability of our findings to current and emerging forms of technological engagement.

Additionally, given the low amount of variation in reports of emotion dysregulation within days, our findings cannot address whether daily technology use is related to within-day variability in adolescents’ reports of their emotional dysregulation. Future studies with more EMA assessment points (to increase temporal granularity) and using methods such as latent profile analysis may be able to assess if adolescents who vary more in emotional dysregulation within-day have different relations with daily technology use than adolescents with little variation.

This study has several strengths. Our use of analytic methods that assess relations within-person allowed for us to use each adolescent as their own control, limiting the impact that confounding external factors may have on the relation between daily technology use and our outcomes (Shiffman et al., 2008). Additionally, our sample of adolescents was representative of the general population in North Carolina’s public school districts at the time of the study and contained a roughly equal amount of boys and girls for the gender moderation analyses.

Our findings have implications for how technology use should be handled for adolescents. Although calls to limit social media and other online activities to protect adolescent mental health continue to grow, the null findings documented in our study do not support a strong link between daily digital technology use and key indicators of well-being.

## Conclusion

Our findings contribute to the growing counter-narrative that technology use does not have a large impact on adolescents’ mental health and well-being. Passive data collection, providing objective and detailed accounts of technology use when supplemented with adolescents’ self-reports, could provide a more full and accurate understanding of adolescents’ online activities and support more definitive evaluations of the relation between use and mental health. Future work should also investigate how some of the newest technologies and platforms, such as the metaverse, impact youth mental health differentially.

## Supplementary Information

Below is the link to the electronic supplementary material.

Supplementary file1 (PPTX 731 KB)

Supplementary file2 (DOCX 20.0 KB)

Supplementary file3 (DOCX 32.1 KB)

Supplementary file4 (DOCX 23.6 KB)

Supplementary file5 (DOCX 12.2 KB)

## Additional Information

### Data Availability

The dataset analyzed during the current study is available upon reasonable request.

### Code Availability

The R code files used to analyze the current study are publicly available under “Files” on Open Science Framework (https://osf.io/fk9zx/).
