# Supplementary figures and images for: Adolescents’ Digital Technology Use, Emotional Dysregulation, and Self-Esteem: No Evidence of Same-Day Linkages
Source: Affect Sci. 2024 Nov 27;5(4):458–67. doi: 10.1007/s42761-024-00282-w (PMC11624161; doi:10.1007/s42761-024-00282-w)

## Slide 1
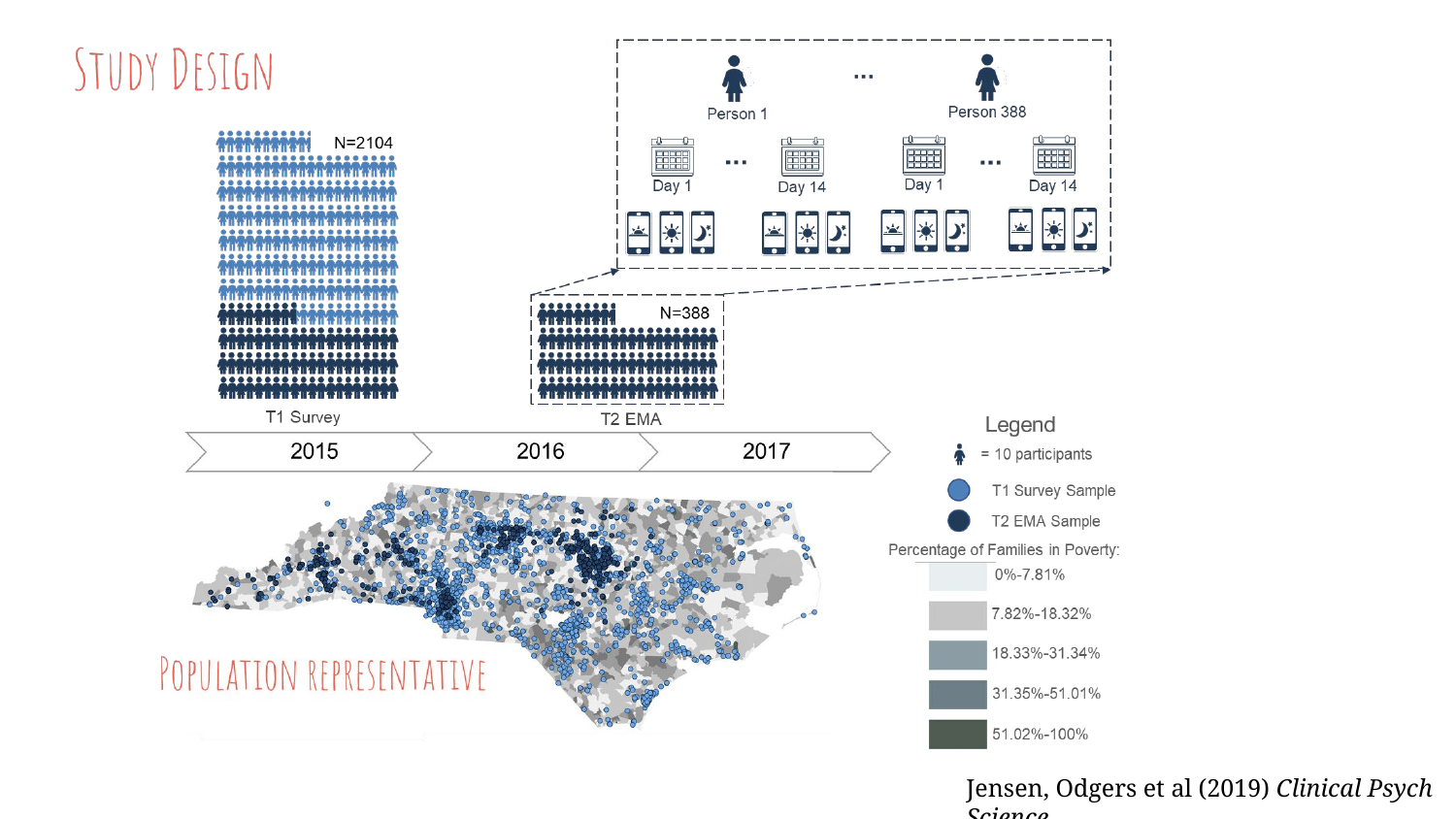

Jensen, Odgers et al (2019) Clinical Psych Science

Supplement: Supplementary file 1 — Supplementary file1 (PPTX 731 KB) [file 42761_2024_282_MOESM1_ESM.pptx]
