# Supplementary material for: Adolescents’ Digital Technology Use, Emotional Dysregulation, and Self-Esteem: No Evidence of Same-Day Linkages
Source: Affect Sci. 2024 Nov 27;5(4):458–67. doi: 10.1007/s42761-024-00282-w (PMC11624161; doi:10.1007/s42761-024-00282-w)
Supplement: Supplementary file 2 — Supplementary file2 (DOCX 20.0 KB) [file 42761_2024_282_MOESM2_ESM.docx]

Supplemental Table 1a. Multilevel Models of Daily Associations between Daily Technology Use for Schoolwork and Emotion Dysregulation

| Predictors | Emotion Dysregulation (Schoolwork)  (4022 days, N=380) | | | | |
| --- | --- | --- | --- | --- | --- |
|  | Estimate | SE | 95%CI |  | p |
| **Fixed** |  |  |  |  |  |
| Intercept | 5.57 | 0.11 | (5.35, 5.79) |  | <0.001 |
| Daily Tech for Schoolwork Hours Within Person | -0.002 | 0.02 | (-0.05, 0.04)* |  | 0.90 |
| Average Tech Hours for Schoolwork(Between person) | 0.38 | 0.10 | (0.19, 0.58)** |  | <0.001 |
| **Random** |  |  |  |  |  |
| Within-person residual variance (σ2) | 0.01 |  |  |  |  |
| Between-person residual variance (τ00) | 4.46 |  |  |  |  |
| ICC | 1.00 |  |  |  |  |

Note: No differences in statistical significance were found between models above and models with first-order autoregressive structure. *Boot CI: (-0.05, 0.04) ** (0.18, 0.58)

Supplemental Table 1b. Multilevel Models of Daily Associations between Daily Technology Use for Social Connection and Emotion Dysregulation

| Predictors | Emotion Dysregulation (Social Connection)  (4021 days, N=378) | | | | |
| --- | --- | --- | --- | --- | --- |
|  | Estimate | SE | 95%CI |  | p |
| **Fixed** |  |  |  |  |  |
| Intercept | 5.59 | 0.11 | (5.36, 5.81) |  | <0.001 |
| Daily Tech for Social Connection Hours Within Person | -0.01 | 0.02 | (-0.05, 0.04)* |  | 0.66 |
| Average Tech Hours for Social Connection (Between person) | 0.10 | 0.05 | (0.003, 0.20)** |  | 0.04 |
| **Random** |  |  |  |  |  |
| Within-person residual variance (σ2) | 0.01 |  |  |  |  |
| Between-person residual variance (τ00) | 4.62 |  |  |  |  |
| ICC | 1.00 |  |  |  |  |

Note: No differences in statistical significance were found between models above and models with first-order autoregressive structure. *Boot CI: (-0.06, 0.03) ** (0.0001, 0.20)

Supplemental Table 1c. Multilevel Models of Daily Associations between Daily Technology Use for Entertainment and Emotion Dysregulation

| Predictors | Emotion Dysregulation (Entertainment)  (4042 days, N=380) | | | | |
| --- | --- | --- | --- | --- | --- |
|  | Estimate | SE | 95%CI |  | p |
| **Fixed** |  |  |  |  |  |
| Intercept | 5.58 | 0.11 | (5.35, 5.80) |  | <0.001 |
| Daily Tech for Entertainment Hours Within Person | -0.06 | 0.02 | (-0.10, -0.02)* |  | 0.01 |
| Average Tech Hours for Entertainment (Between person) | 0.03 | 0.01 | (0.01, 0.06)** |  | 0.01 |
| **Random** |  |  |  |  |  |
| Within-person residual variance (σ2) | 0.03 |  |  |  |  |
| Between-person residual variance (τ00) | 4.57 |  |  |  |  |
| ICC | 0.99 |  |  |  |  |

Note: No differences in statistical significance were found between models above and models with first-order autoregressive structure. *Boot CI: (-0.10, -0.02) ** (0.01, 0.06)

Supplemental Table 1d. Multilevel Models of Daily Associations between Daily Technology Use for Content Creation and Emotion Dysregulation

| Predictors | Emotion Dysregulation (Content Creation)  (4015 days, N= 378) | | | | |
| --- | --- | --- | --- | --- | --- |
|  | Estimate | SE | 95%CI |  | P |
| **Fixed** |  |  |  |  |  |
| Intercept | 5.58 | 0.11 | (5.36, 5.80) |  | <0.001 |
| Daily Tech Hours Within Person for Content Creation | -0.04 | 0.08 | (-0.19, 0.11) |  | 0.62 |
| Average Tech Hours for Content Creation (Between person) | 0.52 | 0.17 | (0.19, 0.85) |  | 0.002 |
| **Random** |  |  |  |  |  |
| Within-person residual variance (σ2) | 0.35 |  |  |  |  |
| Between-person residual variance (τ00) | 4.49 |  |  |  |  |
| ICC | 0.93 |  |  |  |  |

Note: No differences in statistical significance were found between models above and models with first-order autoregressive structure. *Boot CI: (-0.18, 0.11) ** (0.01, 0.06)
