# Supplementary material for: Adolescents’ Digital Technology Use, Emotional Dysregulation, and Self-Esteem: No Evidence of Same-Day Linkages
Source: Affect Sci. 2024 Nov 27;5(4):458–67. doi: 10.1007/s42761-024-00282-w (PMC11624161; doi:10.1007/s42761-024-00282-w)
Supplement: Supplementary file 3 — Supplementary file3 (DOCX 32.1 KB) [file 42761_2024_282_MOESM3_ESM.docx]

Supplemental Table 2a. Multilevel Models of Daily Associations between Technology Use, Emotion Dysregulation, and Self-Esteem with Reduced Dataset ( +/- 2 Standard Deviations Tech Use)

| Predictors | Emotion Dysregulation  (3770 days, N=372) | | | | | Self-Esteem  (3783 days, N=374) | | | | |
| --- | --- | --- | --- | --- | --- | --- | --- | --- | --- | --- |
|  | Estimate | SE | 95%CI |  | p | Estimate | SE | 95%CI |  | p |
| **Fixed** |  |  |  |  |  |  |  |  |  |  |
| Intercept | 5.59 | 0.12 | (5.36, 5.81) |  | <0.001 | 3.94 | 0.04 | (3.86, 4.02) |  | <0.001 |
| Daily Tech Hours Within Person | -0.00 | 0.02 | (-0.07, 0.01)* |  | 0.10 | 0.01 | 0.01 | (-0.004,  0.02) |  | 0.22 |
| Average Tech Hours (Between person) | 0.11 | 0.05 | (0.01, 0.20)** |  | 0.02 | -0.03 | 0.02 | (-0.05,  0.01) |  | 0.13 |
| **Random** |  |  |  |  |  |  |  |  |  |  |
| Within-person residual variance (σ2) | 0.04 |  |  |  |  | 0.001 |  |  |  |  |
| Between-person residual variance (τ00) | 4.63 |  |  |  |  | 0.55 |  |  |  |  |
| ICC | 0.99 |  |  |  |  | 1.00 |  |  |  |  |

Note: No differences in statistical significance were found between models above and models with first-order autoregressive structure. *Boot CI: (-0.0690, 0.0061). ** Boot CI: (0.0197, 0.2055).

Supplemental Table 2b. Multilevel Models of Daily Associations between Technology Use, Emotion Dysregulation, and Self-Esteem with Gender Moderation with Reduced Dataset ( +/- 2 Standard Deviations Tech Use)

| Predictors | Daily Emotion Dysregulation x Gender  (3770 days, N=372) | | | | | Daily Self-Esteem x Gender  (3783 days, N=374) | | | | |
| --- | --- | --- | --- | --- | --- | --- | --- | --- | --- | --- |
|  | Estimate | SE | 95%CI |  | p | Estimate | SE | 95%CI |  | p |
| Fixed |  |  |  |  |  |  |  |  |  |  |
| Intercept | 5.32 | 0.16 | (5.00, 5.64) |  | <0.001 | 4.10 | 0.06 | (3.99, 4.21) |  | <0.001 |
| Within Person | -0.03 | 0.03 | (-0.09, 0.02)* |  | 0.28 | 0.01 | 0.01 | (-0.01, 0.02) |  | 0.50 |
| Between person | 0.01 | 0.06 | (-0.11, 0.14)** |  | 0.85 | -0.003 | 0.02 | (-0.05, 0.04) |  | 0.88 |
| Gender | 0.49 | 0.23 | (0.04, 0.94)*** |  | 0.03 | -0.31 | 0.08 | (-0.46, -0.16) |  | <0.001 |
| Technology X Gender Within | -0.002 | 0.04 | (-0.08, 0.07)**** |  | 0.94 | 0.003 | 0.01 | (-0.02, 0.03) |  | 0.82 |
| Technology x Gender Between | 0.20 | 0.10 | (0.01, 0.39)***** |  | 0.04 | -0.04 | 0.03 | (-0.10, 0.03) |  | 0.27 |
| Random |  |  |  |  |  |  |  |  |  |  |
| Within-person residual variance (σ2) | 4.55 |  |  |  |  | 0.001 |  |  |  |  |
| Between-person residual variance (τ00) | 1.96 |  |  |  |  | 0.53 |  |  |  |  |
| ICC |  |  |  |  |  | 1.00 |  |  |  |  |

Note: No differences in statistical significance were found between models above and models with first-order autoregressive structure for self-esteem. Differences in emotion dysregulation with first-order autoregressive structure are described in text. *Boot CI: (-0.0862, 0.0242) ** (-0.1163, 0.1435 ) *** ( 0.0345, 0.9357 ) **** (-0.0805, 0.0711 ) ***** ( 0.0038, 0.3900).

Supplemental Table 2c. Multilevel Models of Daily Associations between Daily Technology Use for Schoolwork and Emotion Dysregulation with Reduced Dataset ( +/- 2 Standard Deviations Tech Use)

| Predictors | Emotion Dysregulation (Schoolwork)  (3833 days, N=376) | | | | |
| --- | --- | --- | --- | --- | --- |
|  | Estimate | SE | 95%CI |  | p |
| **Fixed** |  |  |  |  |  |
| Intercept | 5.59 | 0.11 | (5.37, 5.81) |  | <0.001 |
| Daily Tech for Schoolwork Hours Within Person | 0.04 | 0.04 | (-0.04, 0.11) |  | 0.36 |
| Average Tech Hours for Schoolwork(Between person) | 0.45 | 0.12 | (0.22, 0.68) |  | <0.001 |
| **Random** |  |  |  |  |  |
| Within-person residual variance (σ2) | 0.10 |  |  |  |  |
| Between-person residual variance (τ00) | 4.58 |  |  |  |  |
| ICC | 0.98 |  |  |  |  |

Supplemental Table 2d. Multilevel Models of Daily Associations between Daily Technology Use for Social Connection and Emotion Dysregulation with Reduced Dataset ( +/- 2 Standard Deviations Tech Use)

| Predictors | Emotion Dysregulation (Social Connection)  (3832 days, N=374) | | | | |
| --- | --- | --- | --- | --- | --- |
|  | Estimate | SE | 95%CI |  | *p* |
| **Fixed** |  |  |  |  |  |
| Intercept | 5.60 | 0.12 | (5.37, 5.83) |  | <0.001 |
| Daily Tech for Social Connection Hours Within Person | -0.03 | 0.04 | (-0.10, 0.04) |  | 0.47 |
| Average Tech Hours for Social Connection (Between person) | 0.12 | 0.06 | (0.001, 0.25) |  | **0.05** |
| **Random** |  |  |  |  |  |
| Within-person residual variance (σ2) | 0.06 |  |  |  |  |
| Between-person residual variance (τ00) | 4.74 |  |  |  |  |
| ICC | 0.99 |  |  |  |  |

Supplemental Table 2e. Multilevel Models of Daily Associations between Daily Technology Use for Entertainment and Emotion Dysregulation with Reduced Dataset ( +/- 2 Standard Deviations Tech Use)

| Predictors | Emotion Dysregulation (Entertainment)  (3853 days, N=376) | | | | |
| --- | --- | --- | --- | --- | --- |
|  | Estimate | SE | 95%CI |  | *p* |
| **Fixed** |  |  |  |  |  |
| Intercept | 5.60 | 0.12 | (5.37, 5.82) |  | <0.001 |
| Daily Tech for Entertainment Hours Within Person | -0.06 | 0.03 | (-0.11, -0.01) |  | 0.03 |
| Average Tech Hours for Entertainment (Between person) | 0.03 | 0.01 | (0.01, 0.06) |  | 0.02 |
| **Random** |  |  |  |  |  |
| Within-person residual variance (σ2) | 0.06 |  |  |  |  |
| Between-person residual variance (τ00) | 4.70 |  |  |  |  |
| ICC | 0.99 |  |  |  |  |

Supplemental Table 2f. Multilevel Models of Daily Associations between Daily Technology Use for Content Creation and Emotion Dysregulation with Reduced Dataset ( +/- 2 Standard Deviations Tech Use)

| Predictors | Emotion Dysregulation (Content Creation)  (3826 days, N= 374) | | | | |
| --- | --- | --- | --- | --- | --- |
|  | Estimate | SE | 95%CI |  | p |
| **Fixed** |  |  |  |  |  |
| Intercept | 5.61 | 0.11 | (5.39, 5.83) |  | <0.001 |
| Daily Tech Hours Within Person for Content Creation | -0.01 | 0.08 | (-0.17, 0.14) |  | 0.90 |
| Average Tech Hours for Content Creation (Between person) | 0.70 | 0.22 | (0.26, 1.14) |  | 0.002 |
| **Random** |  |  |  |  |  |
| Within-person residual variance (σ2) | 0.31 |  |  |  |  |
| Between-person residual variance (τ00) | 4.13 |  |  |  |  |
| ICC | 0.93 |  |  |  |  |
