# Supplementary material for: Adolescents’ Digital Technology Use, Emotional Dysregulation, and Self-Esteem: No Evidence of Same-Day Linkages
Source: Affect Sci. 2024 Nov 27;5(4):458–67. doi: 10.1007/s42761-024-00282-w (PMC11624161; doi:10.1007/s42761-024-00282-w)
Supplement: Supplementary file 4 — Supplementary file4 (DOCX 23.6 KB) [file 42761_2024_282_MOESM4_ESM.docx]

**Supplemental 3: Examining the impact of missing data.**

On average, participants were missing 3 responses for total daily technology use (M = 3.05, SD = 2.95). Quartile 1 had an average of 0.58 missing days (M = 0.58, SD = 0.49), Quartile 2 had an average of 2 missing days (M = 2, SD = 0), Quartile 3 had an average of 3.36 missing days (M = 3.36, SD = 0.48), and Quartile 4 had an average of 7.52 missing days (M = 7.49, SD = 2.76).

Results of the 4 models with missing data as a moderator can be found in Supplemental Tables 3. A statistically significant interaction effect between daily technology use and missing data was detected in only 1 of the 4 models. For adolescents in quartile 4 (i.e., the highest missing days), the within-person effect of daily technology use on emotional dysregulation was more negative when compared to individuals in quartile 1 (i.e., the lowest missing days) (*b* = -0.08, 95%CI (-0.16, -0.002), *p* = 0.04).

Supplemental Table 3a: Multilevel Models of Daily Associations between Technology Use, Emotion Dysregulation, and Self-Esteem with Missingness Moderation

| Predictors | Emotion Dysregulation x Missingness  (3959 days, N=376) | | | | | Self-Esteem x Missingness  (3974 days, N= 377) | | | | |
| --- | --- | --- | --- | --- | --- | --- | --- | --- | --- | --- |
|  | Estimate | SE | 95%CI |  | p | Estimate | SE | 95%CI |  | p |
| Fixed  Intercept | 5.39 | 0.18 | (5.04,  5.74) |  | <0.001 | 3.94 | 0.06 | (3.82, 4.07) |  | <0.001 |
| Within Person | -0.01 | 0.02 | (-0.05,  0.03) |  | 0.53 | 0.00 | 0.01 | (-0.02, 0.01) |  | 0.62 |
| Between person | 0.10 | 0.05 | (0.00, 0.20) |  | 0.05 | -0.03 | 0.02 | (-0.07, 0.01) |  | 0.12 |
| Missing Quartile 2 | -0.31 | 0.33 | (-0.96, 0.35) |  | 0.35 | 0.31 | 0.12 | (0.08, 0.54) |  | 0.01 |
| Missing Quartile 3 | 0.23 | 0.29 | (-0.34, 0.80) |  | 0.43 | -0.14 | 0.10 | (-0.34, 0.06) |  | 0.17 |
| Missing Quartile 4 | 0.84 | 0.31 | (0.24, 1.44) |  | 0.01 | -0.10 | 0.11 | (-0.31, 0.12) |  | 0.37 |
| Between Person Daily Technology Use x Missing Quartile 2 | 0.04 | 0.03 | (-0.02, 0.11) |  | 0.19 | 0.00 | 0.01 | (-0.02, 0.02) |  | 0.96 |
| Between Person Daily Technology Use x Missing Quartile 3 | -0.03 | 0.03 | (-0.09, 0.03) |  | 0.35 | 0.02 | 0.01 | (0.00, 0.04) |  | 0.11 |
| Between Person Daily Technology Use x Missing Quartile 4 | -0.08 | 0.04 | (-0.16, 0.00) |  | 0.04 | 0.02 | 0.01 | (0.00, 0.05) |  | 0.09 |
| Within Person Daily Technology Use x Missing Quartile 2 | -0.15 | 0.11 | (-0.36, 0.06) |  | 0.17 | 0.04 | 0.04 | (-0.04, 0.11) |  | 0.36 |
| Within Person Daily Technology Use x Missing Quartile 3 | -0.05 | 0.08 | (-0.21, 0.11) |  | 0.58 | 0.01 | 0.03 | (-0.05, 0.07) |  | 0.68 |
| Within Person Daily Technology Use x Missing Quartile 4 | 0.02 | 0.09 | (-0.15, 0.19) |  | 0.82 | 0.04 | 0.03 | (-0.02, 0.10) |  | 0.24 |
|  |  |  |  |  |  |  |  |  |  |  |
| **Random** |  |  |  |  |  |  |  |  |  |  |
| Within-person residual variance (σ2) | 0.01 |  |  |  |  | 0.0005 |  |  |  |  |
| Between-person residual variance (τ00) | 4.42 |  |  |  |  | 0.54 |  |  |  |  |
| ICC | 1.00 |  |  |  |  | 1.00 |  |  |  |  |

Supplemental Table 3b: Multilevel Models of Daily Associations between Technology Use, Emotion Dysregulation, and Self-Esteem with Gender and Missingness Moderation

| Predictors | Emotion Dysregulation x Gender X Missingness  (3959 days, N=376) | | | | |  | Self-Esteem x Gender X Missingness  (3974 days, N= 377) | | | | | |
| --- | --- | --- | --- | --- | --- | --- | --- | --- | --- | --- | --- | --- |
|  | Estimate | SE | 95%CI |  | *p* | Estimate | | SE | 95%CI |  |  | *p* |
| **Fixed**  Intercept | 5.11 | 0.21 | (4.70, 5.53) |  | <0.001 | 4.11 | | 0.07 | (3.96, | 4.25) |  | <0.001 |
| Within Person | -0.01 | 0.02 | (-0.05, 0.02) |  | 0.49 | 0.00 | | 0.01 | (-0.01, | 0.01) |  | 0.97 |
| Between person | 0.00 | 0.04 | (-0.08, 0.09) |  | 0.91 | 0.00 | | 0.02 | (-0.03, | 0.03) |  | 0.89 |
| Missing Quartile 2 | -0.22 | 0.32 | (-0.85, 0.42) |  | 0.50 | 0.28 | | 0.11 | (0.06, | 0.51) |  | 0.01 |
| Missing Quartile 3 | 0.19 | 0.29 | (-0.38,  0.76) |  | 0.51 | -0.13 | | 0.10 | (-0.33, | 0.07) |  | 0.22 |
| Missing Quartile 4 | 0.83 | 0.30 | (0.24, 1.42) |  | 0.01 | -0.12 | | 0.11 | (-0.33, | 0.09) |  | 0.26 |
| Gender | 0.52 | 0.22 | (0.08, 0.95) |  | 0.02 | -0.30 | | 0.08 | (-0.46, | -0.15) |  | 0.00 |
| Within Person X Gender | -0.01 | 0.03 | (-0.08, 0.06) |  | 0.75 | 0.00 | | 0.01 | (-0.02, | 0.02) |  | 0.99 |
| Between Person X Gender | 0.14 | 0.08 | (-0.03, 0.30) |  | 0.11 | -0.02 | | 0.03 | (-0.08, | 0.04) |  | 0.57 |
| Between Person Daily Technology Use x Missing Quartile 2 X Gender | 0.05 | 0.05 | (-0.04, 0.15) |  | 0.27 | 0.01 | | 0.02 | (-0.02, | 0.04) |  | 0.50 |
| Between Person Daily Technology Use x Missing Quartile 3 x Gender | -0.03 | 0.04 | (-0.11, 0.06) |  | 0.55 | 0.01 | | 0.01 | (-0.01, | 0.04) |  | 0.31 |
| Between Person Daily Technology Use x Missing Quartile 4 x Gender | -0.05 | 0.06 | (-0.16, 0.06) |  | 0.36 | 0.01 | | 0.02 | (-0.03, | 0.04) |  | 0.62 |
| Within Person Daily Technology Use x Missing Quartile 2 x Gender | -0.16 | 0.18 | (-0.51, 0.20) |  | 0.38 | -0.01 | | 0.06 | (-0.13, | 0.12) |  | 0.91 |
| Within Person Daily Technology Use x Missing Quartile 3 x Gender | -0.07 | 0.10 | (-0.27, 0.14) |  | 0.52 | -0.02 | | 0.04 | (-0.09, | 0.06) |  | 0.67 |
| Within Person Daily Technology Use x Missing Quartile 4 x Gender | 0.28 | 0.15 | (0.00, 0.57) |  | 0.05 | -0.01 | | 0.05 | (-0.11, | 0.08) |  | 0.78 |
| **Random** |  |  |  |  |  |  | |  |  |  |  |  |
| Within-person residual variance (σ2) | 0.01 |  |  |  |  | 0.001 | |  |  |  |  |  |
| Between-person residual variance (τ00) | 4.25 |  |  |  |  | 0.52 | |  |  |  |  |  |
| ICC | 1.00 |  |  |  |  | 1.00 | |  |  |  |  |  |
