# Supplementary material for: Adolescents’ Digital Technology Use, Emotional Dysregulation, and Self-Esteem: No Evidence of Same-Day Linkages
Source: Affect Sci. 2024 Nov 27;5(4):458–67. doi: 10.1007/s42761-024-00282-w (PMC11624161; doi:10.1007/s42761-024-00282-w)
Supplement: Supplementary file 5 — Supplementary file5 (DOCX 12.2 KB) [file 42761_2024_282_MOESM5_ESM.docx]

Click here to download Link(s) to supporting data

<http://doi.org/10.17605/OSF.IO/4J2P8>
